# The First Complete Genome Sequences of Hepatitis C Virus Subtype 2b from Latin America: Molecular Characterization and Phylogeographic Analysis

**DOI:** 10.3390/v11111000

**Published:** 2019-10-31

**Authors:** Natália Spitz, José J. Barros, Kycia M. do Ó, Carlos E. Brandão-Mello, Natalia M. Araujo

**Affiliations:** 1 Laboratory of Molecular Virology, Oswaldo Cruz Institute, FIOCRUZ, Rio de Janeiro RJ 21040-360, Brazil; natalia.toledo@ioc.fiocruz.br (N.S.); barros@ioc.fiocruz.br (J.J.B.); 2 Viral Hepatitis Advisory Committee of the Ministry of Health, Brasilia DF 70058-900, Brazil; kyciadoo@gmail.com; 3 Gaffrée & Guinle Universitary Hospital, Federal University of Rio de Janeiro State, UNIRIO, Rio de Janeiro RJ 20270-901, Brazil; cedubrandao@gmail.com

**Keywords:** hepatitis C virus, HCV subtypes, Latin America, RT-PCR, full-length genome, Bayesian framework, phylogeography

## Abstract

The hepatitis C virus (HCV) has remarkable genetic diversity and exists as eight genotypes (1 to 8) with distinct geographic distributions. No complete genome sequence of HCV subtype 2b (HCV-2b) is available from Latin American countries, and the factors underlying its emergence and spread within the continent remain unknown. The present study was conducted to determine the first full-length genomic sequences of HCV-2b isolates from Latin America and reconstruct the spatial and temporal diversification of this subtype in Brazil. Nearly complete HCV-2b genomes isolated from two Brazilian patients were obtained by direct sequencing of long PCR fragments and analyzed together with reference sequences using the Bayesian coalescent and phylogeographic framework approaches. The two HCV-2b genomes were 9318 nucleotides (nt) in length (nt 37–9354). Interestingly, the long RT-PCR technique was able to detect co-circulation of viral variants that contained an in-frame deletion of 2022 nt encompassing E1, E2, and p7 proteins. Spatiotemporal reconstruction analyses suggest that HCV-2b had a single introduction in Brazil during the early 1980s, displaying an epidemic history characterized by a low and virtually constant population size until the present time. These results coincide with epidemiological data in Brazil and may explain the low national prevalence of this subtype.

## 1. Introduction

The hepatitis C virus (HCV) is a single-stranded positive-sense RNA virus belonging to the *Flaviviridae* family and is the leading etiologic agent of chronic liver disease [1]. According to the WHO, an estimated 71 million people worldwide suffer from chronic hepatitis C infection, resulting in 399,000 deaths mainly attributable to cirrhosis and hepatocellular carcinoma [2]. The HCV genome, which is approximately 9600 nucleotides (nt) long, contains two short untranslated regions at each end (5’UTR and 3’UTR) along with a single open reading frame encoding three structural (core, E1, and E2) and seven non-structural (p7, NS2, NS3, NS4A, NS4B, NS5A, and NS5B) proteins. 

The high rate of replication (up to 10^12^ particles produced per day) coupled with an error-prone mechanism results in the extremely high sequence diversity of HCV [3]. In particular, HCV variants characterized by large in-frame genomic deletions typically encompassing E1–NS2 regions have been detected in patient sera and liver tissues [4,5,6,7,8,9]. These subgenomic HCV species, believed to be generated from the full-length viral genome through polymerase errors, can be transpackaged into infectious virions in the presence of a wild-type virus [5]. So far, their biological roles are yet to be established. 

Due to accumulating mutations during the natural course of history, HCV isolates are classified into eight genotypes (1 to 8) and numerous subtypes [10,11]. The official classification of a new HCV type minimally requires one complete genome sequence to differ from other sequences by at least 30% (genotype) or 15% (subtype) [11,12]. Despite the revolutionary progress recently made with direct-acting antivirals (DAAs) against HCV, identification of the HCV genotype/subtype remains an important requirement for effective treatment decisions and serves as a good predictor of treatment success [13,14]. Therefore, HCV whole-genome sequencing appears useful for the accurate determination of viral genotypes and resistance-associated substitutions throughout the DAA target genome regions (NS3, NS5A, and NS5B) as well as identifying recombinant or rare viral types [15,16,17].

The global distribution of HCV isolates is characterized by regional variations in genotype/subtype prevalence and potentially influenced by historical and contemporary trends in human migration. HCV subtypes 1a, 1b, 2a, 2b, 2c, and 3a have worldwide distribution and account for a large proportion of HCV infections. On the other hand, many of the other HCV subtypes are comparatively rare and circulate in more restricted geographical areas such as endemic strains from genotypes 1 and 2 in West Africa, 3 in South Asia, 4 in Central Africa and the Middle East, 5 in Southern Africa, 6 in South East Asia, 7 in Canada [18,19], and 8 in India [10].

While HCV genotype 1 is the most prevalent genotype in the Latin American region, genotype 2 isolates have been successful in terms of establishment and dissemination in different American countries [20,21]. Specifically, HCV subtype 2b (HCV-2b) has been detected throughout the Caribbean (Martinique) and Central (Mexico) and South (Brazil, Colombia and Venezuela) America, as determined based on the HCV sequences reported to the Los Alamos database (https://hcv.lanl.gov/content/sequence/HCV/ToolsOutline.html). However, no complete genome sequences of HCV-2b are available from these countries, limiting the contribution of Latin American isolates to phylogenetic and phylogeographic studies. Moreover, the evolutionary history of HCV-2b in Brazil has not been investigated. Accordingly, the main objectives of the current study were to determine the first full-length HCV-2b genomes from Latin America and reconstruct the spatial and temporal diversification of this subtype in Brazil.

## 2. Materials and Methods

### 2.1. Patients

HCV-2b isolates were obtained from two patients referred to the Gaffrée & Guinle University Hospital in Rio de Janeiro, Brazil. Patient 1 was a 59 year-old woman serologically positive for anti-HCV since 2018 with a METAVIR fibrosis score F0 (no fibrosis) and no prior history of HCV treatment. Patient 2 was an 81 year-old woman serologically positive for anti-HCV since 1999 with a METAVIR fibrosis score F4 (cirrhosis) and no current HCV treatment. This patient had a prior history of non-response to interferon alfa-2a and ribavirin combination therapy. The study protocol was approved by the Ethics Committee of Oswaldo Cruz Institute (no. 2.927.747) and informed consent was obtained from both patients.

### 2.2. Long Template RT-PCR and Genome Sequencing

**Figure 1:**
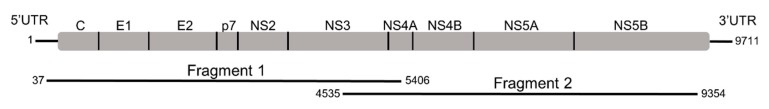
Generalized approach to amplify the full-length HCV genome.

The RT-PCR assay was optimized to amplify the nearly complete genome of HCV-2b via two long PCR fragments (fragment 1 (nt 37 to 5406) and fragment 2 (nt 4535 to 9354); Figure 1).

For each genomic fragment, total RNA was extracted from 200 μL serum using a High Pure Viral Nucleic Acid Kit (Roche Diagnostics, Mannheim, Germany) according to the manufacturer’s instructions. Eluted RNA was precipitated at −20 °C overnight with 0.10 volumes of 3 M sodium acetate and 2.5 volumes of absolute ethanol and resuspended in 9.5 μL diethyl pyrocarbonate (DEPC)-treated water. For reverse transcription of HCV RNA, the total volume of precipitated RNA (9.5 μL) was incubated with 0.5 mM dNTPs and 0.1 μM anti-sense primer HCV5423R (fragment 1) and HCV9373R (fragment 2) (Table 1) at 65 °C for 5 min and subsequently placed on ice for 3 min. Next, cDNA synthesis was conducted by adding 200 U SuperScript IV Reverse Transcriptase, 4 μL 5X SSIV Buffer, 5 mM DTT, and 2 U RNaseOUT RNase Inhibitor (Invitrogen, Carlsbad, CA, USA) to the mixture and incubating at 53 °C for 10 min. The reaction was inactivated at 80 °C for 10 min, followed by the addition of 2 U of RNase H (Invitrogen) at 37 °C for 20 min for RNA removal. Special care was taken during all the mixing steps to avoid shearing of the long RNA template.

Amplification of the two genomic fragments was performed using nested PCR. The first round of PCR was carried out in a 25 μL reaction volume containing 3 μL cDNA, 0.2 μM outer primers HCV17S and HCV5423R (fragment 1), HCV4328S and HCV9373R (fragment 2) (Table 1), 2.5 μL 10X High Fidelity PCR Buffer (Invitrogen), 2 mM MgSO_4_, 0.2 mM dNTPs, 1 μL dimethyl sulfoxide (DMSO) (Life Technologies, Carlsbad, CA, USA), and 0.5 U Platinum *Taq* DNA Polymerase High Fidelity (Invitrogen). The second round of PCR was performed in a 50 μL reaction volume consisting of 2 μL of the first assay product, 0.2 μM inner primers HCV37S and HCV5406R (fragment 1) and HCV4535S and HCV9354R (fragment 2) (Table 1), 5 μL 10× High Fidelity PCR Buffer (Invitrogen), 2.0 mM MgSO_4_, 0.2 mM dNTPs, 2 μL DMSO (Life Technologies), and 1 U Platinum *Taq* DNA Polymerase High Fidelity (Invitrogen). Thermal conditions for both PCR assays included an initial denaturation step at 94 °C for 2 min, 35 cycles at 94 °C for 30 s, 55 °C for 30 s, and 68 °C for 5 min, and a final extension step at 68 °C for 10 min. PCR products were resolved on 1% agarose gel and purified with the Wizard SV Gel and PCR Clean-Up System (Promega, Madison, WI, USA). HCV full-length genome sequences were determined via direct sequencing using a BigDye Terminator Kit v3.1 (Applied Biosystems, Foster City, CA, USA) and a set of 17 specific primers distributed along the HCV genome (Table 1). Sequencing reactions were analyzed on an ABI3730xl automated sequencer (Applied Biosystems).

### 2.3. Phylogenetic and Genetic Analyses

Multiple sequence alignment was performed using the MUSCLE program and subjected to Maximum Likelihood (ML) phylogenetic analysis. ML phylogenetic trees were inferred with the online version of the PhyML program [22] under the GTR + I + G nucleotide substitution model selected with SMS (Smart Model Selection in PhyML) [23]. A heuristic tree search was performed with the aid of the SPR branch-swapping algorithm and the reliability of phylogeny estimated with the approximate likelihood-ratio test [24] based on a Shimodaira–Hasegawa-like procedure (SH-aLRT). To identify possible recombination, bootscan analyses of full-length sequences were performed in the SimPlot software program version 3.5.1 [25] with a sliding window size of 200 bp and step size of 20 bp increment. Potential DAA resistance-associated substitutions among the NS3, NS5A, and NS5B genomic regions were investigated using the Geno2pheno[HCV] interpretation system [26]. HCV reference isolate HC-J6CH (genotype 2; GenBank accession number NC_009823) was used for numbering the nucleotides, amino acids, and deletions [27]. Mean genetic distances between groups of full-length HCV subtype 2b sequences from different geographical regions were estimated via Kimura 2-parameter analysis.

### 2.4. HCV-2b Datasets

Two datasets containing HCV-2b sequences retrieved from public databases (Los Alamos and GenBank) were constructed. Full listings of accession numbers and sampling locations are provided in Table S1. Dataset 1 comprised 181 partial NS5B sequences (353 nt, nucleotide positions 8694–9046) with known country of origin as well as the two genomes sequenced in this study. As sampling dates were not available for all sequences included in dataset 1, a smaller dataset (dataset 2) specifically comprising isolates with known collection dates (*n* = 134) was created. Sequences were aligned using MUSCLE software [28]. The phylogenetic signal of the aligned nucleotide sequences from datasets 1 and 2 was examined using a test of substitution saturation [29] implemented in the DAMBE7 program [30].

### 2.5. Molecular Clock Analysis

The substitution rate (nucleotide substitutions per site per year, s/s/y) and time of the most recent common ancestor tMRCA (years) were inferred based on sequences sampled at different time-points (dataset 2) using a Bayesian Markov Chain Monte Carlo (MCMC) approach implemented in BEAST v1.10.4 [31] along with BEAGLE v3.1 to improve run time [32]. The temporal structure of the dataset was assessed by conducting regression of root-to-tip genetic distances against year of sampling using TempEst v1.5 [33]. Analyses were performed using the GTR + I + G nucleotide substitution model, which was the optimal model selected using the JModelTest [34,35] and the Bayesian Skyline coalescent tree prior [36]. The most suitable clock model was selected after running the analysis separately using strict and relaxed (uncorrelated lognormal) clocks. MCMC was run for 400 million generations with sampling every 40,000 generations. The effective sample size (ESS) value for each parameter was >100, indicating sufficient mixing of the Markov chain.

### 2.6. Bayesian Phylogeographic Analyses

Spatial reconstruction was achieved by applying a reversible discrete Bayesian phylogeographic model [37] using dataset 1. The Bayesian stochastic search variable selection (BSSVS) model was implemented, which allows a zero diffusion rate with a positive prior probability. Uncertainty of parameter estimates was assessed after excluding the initial 10% of the run by calculating the 95% Highest Probability Density (HPD) values using the TRACER v1.7.1 program. Maximum clade credibility (MCC) trees were summarized from the posterior distribution of trees with TreeAnnotator and visualized with FigTree v1.4.3.

## 3. Results and Discussion

Here, we successfully optimized the RT-PCR assay to amplify long fragments of the HCV-2b genome. HCV whole-genome sequencing is technically challenging and most assays described to date use several overlapping amplicons [17], which is time-consuming and increases selective bias due to the use of multiple primers. With the methodology described in this study, near-full-length genome sequences of HCV-2b were obtained by direct sequencing of only two overlapping amplicons (Figure 1).

**Figure 2:**
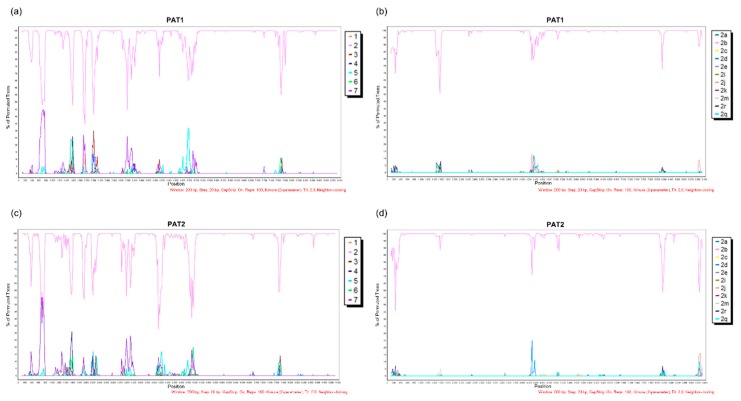
Analysis of potential recombination events of the PAT1 and PAT2 isolates. (**a**) BootScan plot of the PAT1 and HCV genotypes. (**b**) BootScan plot of the PAT1 and HCV-2 subtypes. (**c**) BootScan plot of the PAT2 and HCV genotypes. (**d**) BootScan plot of the PAT2 and HCV-2 subtypes. The parameters used for analysis are shown at the bottom of the figures. All analyses were performed within a window of 200 bp and step size of 20 bp under the Kimura 2-parameter model.

The complete genome of isolate PAT1 (from patient 1) consisted of 9318 nt excluding the polypyrimidine tract, with a G + C content of 56.2% harboring the 10 HCV genomic regions. The complete PAT1 sequence has been deposited in the GenBank database under the accession number MN385563. Bootscan analysis reflected no evidence of recombination between different HCV genotypes (Figure 2a) or HCV-2 subtypes (Figure 2b). Moreover, no potential DAA resistance-associated substitutions were detected in PAT1.

To establish whether our RT-PCR assay could amplify other HCV-2b isolates, serum sample from a second chronic hepatitis C patient (patient 2) was included for study. Unexpectedly, a deletion of 2022 nt (genome positions 964–2985) encompassing E1, E2, p7, and the 5’ end of NS2 genomic regions was observed in one of the amplicons. A PCR primer pair (2bDel1 and HCV3007R) flanking the deleted region was used to investigate co-infection with wild-type isolates (Table 1). Using this strategy, we were able to amplify the deleted genomic region and obtain the complete genome of the PAT2 isolate. Patient 2 was an 81 year-old cirrhotic patient, corroborating earlier findings of an association of HCV large in-frame deletion mutants with patient age and increased necroinflammatory activity in the liver [5]. Previous reports have demonstrated a prevalence of these mutants in 19–26% of infected patients and proposed a potential role in viral persistence [5,6]. PAT2 contained 9318 nt excluding the polypyrimidine tract, with a G + C content of 55.8% harboring the 10 HCV genomic regions. The complete genome sequence of PAT2 has been deposited in the GenBank database under the accession number MN385564. Bootscan analysis revealed no evidence of recombination between different HCV genotypes (Figure 2c) or HCV-2 subtypes (Figure 2d). No potential DAA resistance-associated substitution was detected in PAT2.

**Figure 3:**
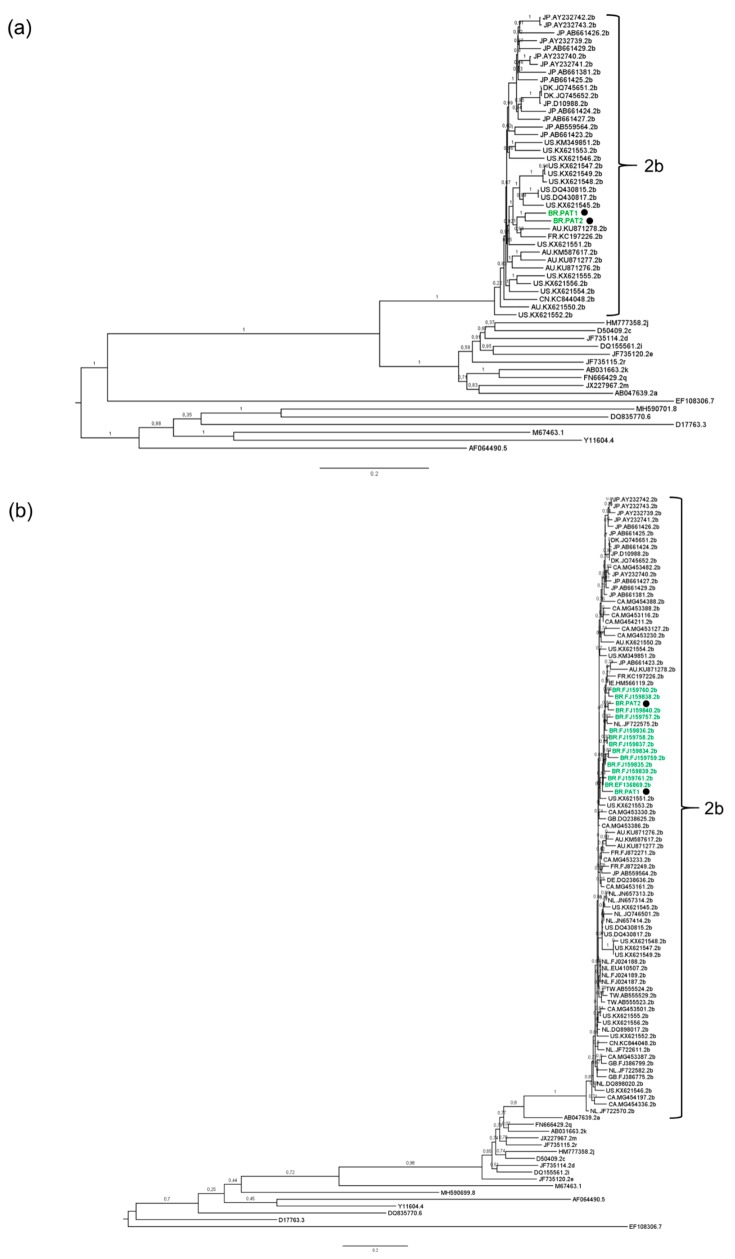
Phylogenetic analysis of HCV sequences. Brazilian HCV sequences are depicted in green. The sequences generated in this study are highlighted with the symbol ●. Reference sequences are indicated by the accession number followed by subtype. The numbers in branches indicate statistical support (aLRT value). (**a**) Maximum Likelihood (ML) phylogenetic tree of HCV complete genome sequences. (**b**) ML phylogenetic tree of HCV NS5B sequences.

Phylogenetic reconstructions based on complete genome and partial NS5B sequences of representative HCV genotypes 1 to 8 led to the classification of PAT1 and PAT2 isolates as HCV-2b (Figure 3a,b). Interestingly, PAT1 and PAT2 clustered in a well-supported monophyletic clade (aLRT = 0.84) together with all Brazilian NS5B HCV-2b sequences (Figure 3b). The mean genetic distance between our two Brazilian full-length HCV-2b genome sequences and 105 HCV-2b genomes from other regions worldwide was additionally calculated (Table 2). Comparable values (0.079–0.107) were obtained among all geographical groups analyzed (Australia, Brazil, China, Denmark, France, Japan, and the USA), indicating that HCV-2b is genetically similar among global isolates (Table 2). In fact, HCV epidemic subtypes such as HCV-2b are characterized by low genetic diversity, high prevalence, and a global distribution. On the other hand, endemic strains are more spatially restricted but harbor greater genetic diversity due to low transmission rates and their centuries-long persistence in geographically restricted areas [38,39].

For a more comprehensive analysis of the time and epicenter of diversification of HCV-2b in Brazil, a Bayesian MCMC analysis was conducted on dataset 1 (181 partial NS5B sequences from databanks and two from this study). Datasets 1 and 2 showed the appropriate phylogenetic signal for consistent phylogenetic and molecular clock inferences, since no substitution saturation was detected using either the test developed by Xia et al. (2003) [29] (Table S2) or transition and transversion versus divergence graphics (Supplemental Figure S1). Dataset 2 had a temporal structure, as revealed by the positive correlation coefficient between genetic divergence and time (Supplemental Figure S2), indicating that the timescale of HCV could be directly estimated from sampling dates of selected sequences. The analysis under the strict molecular clock model showed no convergence, even after combining two independent runs with 400 million states. Under the relaxed clock model, the estimated mean substitution rate of HCV-2b NS5B was 1.06 × 10^−3^ (95% HPD: 4.87 × 10^−4^–1.66 × 10^−3^) s/s/y, similar to previously estimated NS5B gene substitution rates [40]. The coefficient of rate variation for HCV-2b was significantly higher than zero, supporting the use of the relaxed molecular clock model.

**Figure 4:**
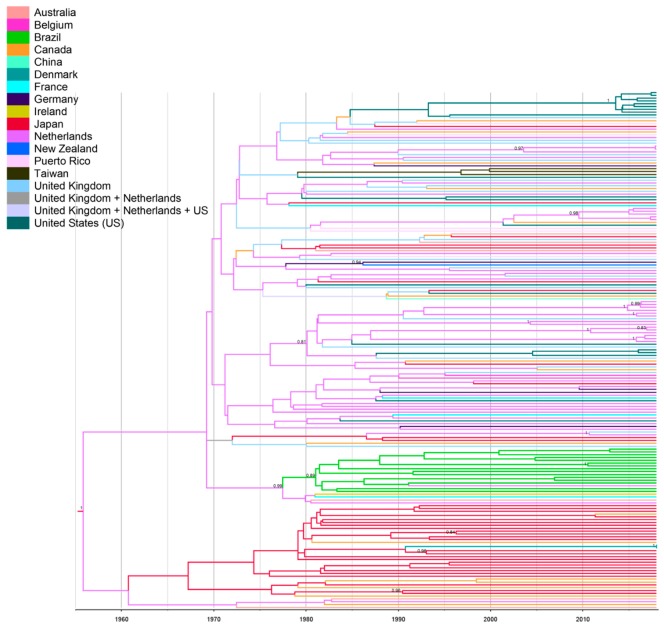
Bayesian maximum clade credibility tree of HCV-2b NS5B sequences. Branches are colored according to the potential locations of the parental node (colored legends in the figure). The scale at the bottom of the tree represents the years before the last sampling time. The numbers on the internal nodes represent posterior probabilities (pp). The tree was automatically rooted under the assumption of a relaxed molecular clock.

**Figure 5:**
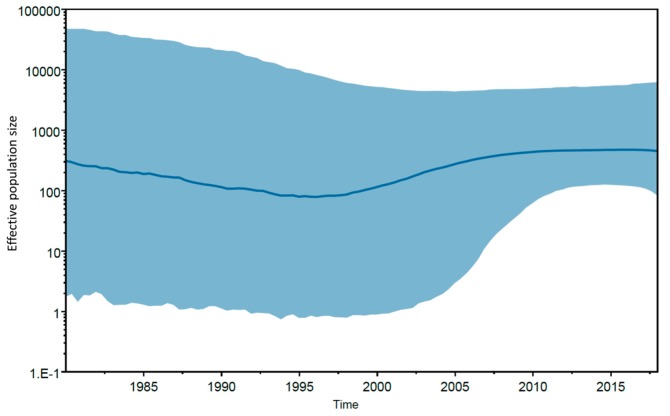
Bayesian skyline plot showing the epidemic history of Brazilian HCV-2b. Median (solid line) and upper and lower 95% HPD (solid area) estimates of the effective population size (*Y*-axis; log_10_ scale) through time (*X*-axis; calendar years) are presented in the graph.

Data from spatiotemporal reconstruction analysis showed that HCV-2b Brazilian sequences grouped into a highly supported monophyletic cluster (posterior probability (PP) = 0.89), indicating a single introduction of this subtype into the country. The Netherlands was the most probable source of HCV-2b introduction into Brazil (PSP = 0.31), which may have occurred during the early 1980s (tMRCA = 1982; 95% HPD: 1976–1990) (Figure 4). Notably, HCV subtypes 2a and 2b account for 10% of chronic HCV infections in the Netherlands [41]. The increase in parenteral routes of transmission such as contaminated blood products, transfusion, injecting drug use, and invasive medical procedures during the 20th century resulted in global dispersal of the current epidemic subtypes including HCV-2b [38,39,42]. Interestingly, the Bayesian skyline plot of Brazilian HCV-2b showed a low and virtually constant population size lasting until the present time (Figure 5). Expansion of HCV-2b in Brazil might have been hampered by the measures applied to prevent parenteral transmission of other viral agents such as HBV and HIV during the 1980s, followed by the introduction of anti-HCV screening tests in blood banks in 1993. These results corroborate previous reports [43] that the growth rate of the major HCV subtypes 1a, 1b, and 3a in Brazil has decreased since 1980–1995, suggesting that expansion of HCV may have been effectively controlled. Furthermore, according to Lampe et al. (2010) [43], the main Brazilian HCV subtypes potentially started to circulate within the country after 1940, with multiple independent introductions of each subtype into the population. Conversely, our findings suggest a single and more recent introduction of HCV-2b in Brazil, which might explain the lower prevalence of this subtype countrywide [44].

In conclusion, the long RT-PCR assay developed in this study facilitated the determination of the first complete genome sequences of HCV-2b from Latin America, increasing the contribution of Latin American isolates to ongoing phylogenetic and phylogeographic studies. Moreover, HCV-2b displayed a different epidemic history from the major Brazilian subtypes characterized by a relatively late and unique introduction into the country during the early 1980s. Elucidation of the history of the spread of HCV and associated epidemiological processes should be helpful in improving targeted HCV prevention and screening campaigns, and ultimately, the development of novel strategies to eliminate HCV.

## Figures and Tables

**Table 1:** Primers used for amplification and sequencing of the HCV genome.

Primer	Primer Sequence 5′–3′	Sense	Position ^a^
RT-nested-PCR primers			
HCV17S	GGCGACACTCCGCCATGAATCACT	forward	17–40
HCV37S	CACTCCCCTGTGAGGAACTACTGTCTTCACG	forward	37–67
2bDel1	CTCSAACARCAGCATYACYTGGC	forward	961–983
HCV3007R	CACHAGGCGTGGGTGBAGAATG	reverse	3007–2986
HCV4328S	ATCCTTGGCATTGGAACRGTCCTYGACC	forward	4328–4355
HCV4535S	CATTCAAAGAAGAAGTGCGAYGAGCT	forward	4535–4560
HCV5406R	CGGCCDATGATGGAAAYGCAGCC	reverse	5406–5384
HCV5423R	GATCATTCAGGTGTADGCGGCC	reverse	5423–5402
HCV9354R	CTGTGAWADATGTCGCCCCCG	reverse	9354–9334
HCV9373R	GGGTCGGGCATGCGACACGCTGTGAWADATGTC	reverse	9373–9341
Sequencing primers			
HCV37S	CACTCCCCTGTGAGGAACTACTGTCTTCACG	forward	37–67
S7	AGACCGTGCACCATGAGCAC	forward	329–348
A5	TACGCCGGGGGTCAKTRGGGCCCCA	reverse	683–659
HCV944S	TACGCCACYAATGATTGCTC	forward	944–963
Del1597	CTGGCACATAAATCGGACCG	forward	1597–1616
HCV3007S	CATTCTVCACCCACGCCTDGTG	forward	3007–2986
HCV3920S	GCCAARTCYATTGACTTCATCCC	forward	3920–3942
HCV4356R	TGGTCRAGGACYGTTCCRATGCC	reverse	4356–4334
HCV4535	CATTCAAAGAAGAAGTGCGAYGAGCT	forward	4535–4560
HCV5285S	ATCGCCACGTGCATGCARGCT	forward	5285–5305
HCV5406R	CGGCCDATGATGGAAAYGCAGCC	reverse	5406–5384
HCV6339R	GACAGCCAGTTYTTRAAGTCTG	reverse	6339–6318
HCV6984S	TGAAGGCYACCTGYACCACYCA	forward	6984–7005
HCV7059R	TCRCCYCCCATGAAVAGRTT	reverse	7059–7040
PR4	GCNGARTAYCTVGTCATAGCCTC	reverse	8709–8687
HCV9354R	CTGTGAWADATGTCGCCCCCG	reverse	9354–9334
HCV9140S	CTTGGAGCGCCTCCCCTYAG	forward	9140–9159

^a^ With reference to numbering nucleotides of HCV-2a (HC-J6CH).

**Table 2:** Mean genetic distances between groups of full-length HCV subtype 2b sequences from different geographical regions.

	Brazil	France	USA	Japan	Australia	China	Denmark
**Brazil**		0.003	0.002	0.003	0.003	0.003	0.003
**France**	0.090		0.002	0.003	0.002	0.004	0.004
**USA**	0.100	0.097		0.002	0.002	0.003	0.002
**Japan**	0.106	0.102	0.098		0.002	0.003	0.002
**Australia**	0.103	0.101	0.099	0.101		0.003	0.003
**China**	0.105	0.102	0.101	0.098	0.101		0.004
**Denmark**	0.107	0.101	0.095	0.079	0.100	0.097	

The gray values represent the standard deviation.

## Supplementary Materials

The following are available online at https://www.mdpi.com/1999-4915/11/11/1000/s1. Figure S1: Transition and transversion versus divergence graphics performed for nucleotide sequence alignments. The transitions (s, blue) and transversions (v, green) are plotted against genetic distance according to the general time reversible (GTR) model. (a) Dataset 1. (b) Dataset 2. Figure S2: Root-to-tip regression analysis of temporal structure. Plot of the root-to-tip genetic distance against sampling time is shown for phylogeny estimated from the alignment. Each dot represents a sequence. R^2^ value is given as an indicator of the degree to which evolution has been clock-like. Table S1: GenBank accession numbers of the 181 partial NS5B HCV-2b sequences used in the Bayesian phylogeographic analysis. Table S2: Index of substitution rate (Iss) and critical index of substitution rate (Iss.c) of the NS5B sequence datasets used for Bayesian analysis.
