# Supplementary material for: The First Complete Genome Sequences of Hepatitis C Virus Subtype 2b from Latin America: Molecular Characterization and Phylogeographic Analysis
Source: Viruses. 2019 Oct 31;11(11):1000. doi: 10.3390/v11111000 (PMC6893431; doi:10.3390/v11111000)
Supplement: Supplementary file 1 [file viruses-11-01000-s001.zip › Table S2.docx]

**Table S2.** Index of substitution rate (Iss) and critical index of substitution rate (Iss.c) of the NS5B sequence datasets used for Bayesian analysis.

| Dataset | I_SS_* | I_SS.C_* | P(two-tailed t-test) |
| --- | --- | --- | --- |
| 1 | 0.108 | 0.686 | <0.001 |
| 2 | 0.104 | 0.686 | <0.001 |

*Values based on 32 operational taxonomic units randomly selected.
