# Supplementary material for: The First Complete Genome Sequences of Hepatitis C Virus Subtype 2b from Latin America: Molecular Characterization and Phylogeographic Analysis
Source: Viruses. 2019 Oct 31;11(11):1000. doi: 10.3390/v11111000 (PMC6893431; doi:10.3390/v11111000)
Supplement: Supplementary file 1 [file viruses-11-01000-s001.zip › Table S1.docx]

**Table S1.** GenBank accession numbers of the 181 NS5B HCV-2b sequences used in the Bayesian phylogeographic analysis.

| Country | Accession numbers |
| --- | --- |
| Australia | KM587617, KU871276, KU871277, KU871278, KX621550, MG454601 |
| Belgium | KX346813, KX346814 |
| Brazil | EF136869, FJ159757, FJ159758, FJ159759, FJ159760, FJ159761, FJ159834, FJ159835, FJ159836, FJ159837, FJ159838, FJ159839, FJ159840 |
| Canada | MG453116, MG453127, MG453161, MG453230, MG453233, MG453330, MG453386, MG453387, MG453388, MG453482, MG453501, MG454197, MG454211, MG454336, MG454388 |
| China | KC844048 |
| Germany | DQ238636,MG453295, MG453336, MG453337, MG454212 |
| Denmark | JQ745651, JQ745652 |
| France | FJ872249, FJ872271, KC197226, MG453244 |
| United Kingdom | DQ238625, FJ386775, FJ386799, MG453155, MG453169, MG453171, MG453187, MG453188, MG453190, MG453239, MG453256, MG453288, MG453327, MG453355, MG453361, MG453404, MG453466, MG453489, MG453497, MG454150, MG454162, MG454171 |
| Ireland | HM566119 |
| Japan | AB559564, AB661373, AB661374, AB661375, AB661377, AB661378, AB661379, AB661380, AB661382, AB661383, AB661384, AB661385, AB661386, AB661388, AB661390, AB661391, AB661394, AB661395, AB661396, AB661401, AB661406, AB661407, AB661408, AB661411, AB661412, AB661413, AB661414, AB661415, AB661416, AB661419, AB661422, AB661423, AB661430, AY232732, AY232746, D00828 |
| Netherlands | DQ898017, DQ898020, EU410507, FJ024187, FJ024188, FJ024189, FJ024190, FJ024191, JF722457, JF722458, JF722459, JF722469, JF722474, JF722483, JF722485, JF722493, JF722495, JF722499, JF722502, JF722504, JF722513, JF722516, JF722520, JF722527, JF722532, JF722538, JF722549, JF722570, JF722575, JF722579, JF722582, JF722611, JN657313, JN657314, JN657336, JN657337, JN657397, JN657398, JN657399, JN657412, JN657413, JN657414, JQ746501, KY386883 |
| New Zealand | MG454355 |
| Puerto Rico | MG453632, MG453634, MG453711, MG453724, MG453731 |
| Taiwan | AB555523, AB555524, AB555529 |
| United States | EF523596, JQ061496, JQ061497, JQ061498, JQ061499, JQ061500, JQ061501, JQ061502, JQ061503, KM349851, KX621545, KX621546, KX621547, KX621548, KX621549, KX621551, KX621552, KX621553, KX621554, KX621555, KX621556 |
