# Supplementary material for: The First Complete Genome Sequences of Hepatitis C Virus Subtype 2b from Latin America: Molecular Characterization and Phylogeographic Analysis
Source: Viruses. 2019 Oct 31;11(11):1000. doi: 10.3390/v11111000 (PMC6893431; doi:10.3390/v11111000)
Supplement: Supplementary file 1 [file viruses-11-01000-s001.zip › BR_sequences.docx]

>PAT1

CACTCCCCTGTGAGGAACTACTGTCTTCACGCAGAAAGCGTCTAGCCATGGCGTTAGTATGAGTGTCGTACAGCCTCCAGGCCCCCCCCTCCCGGGAGAGCCATAGTGGTCTGCGGAACCGGTGAGTACACCGGAATTACCGGAAAGACTGGGTCCTTTCTTGGATAAACCCACTCTATGTCCGGTCATTTGGGCGTGCCCCCGCAAGACTGCTAGCCGAGTAGCGTTGGGTTGCGAAAGGCCTTGTGGTACTGCCTGATAGGGTGCTTGCGAGTGCCCCGGGAGGTCTCGTAGACCGTGCATCATGAGCACAAATCCTAAACCTCAAAGAAAAACCAAAAGAAACACAAACCGCCGCCCACAGGACGTTAAGTTCCCGGGTGGCGGCCAGATCGTTGGCGGAGTTTACTTGCTGCCGCGCAGGGGCCCCAGGTTGGGTGTGCGCGCGACAAGGAAGACTTCCGAGCGATCCCAGCCGCGTGGGAGACGCCAGCCCATCCCGAAAGATCGGCGCTCCACCGGCAAGTCCTGGGGAAAGCCAGGATACCCTTGGCCCCTGTATGGAAACGAGGGCTGCGGTTGGGCAGGTTGGCTCCTGTCCCCCCGCGGTTCGCGTCCTACTTGGGGCCCCACTGACCCCCGGCATAGATCACGCAACTTGGGCAAAGTCATCGACACCATTACGTGTGGTTTTGCCGACCTCATGGGGTACATCCCGGTCGTTGGCGCCCCGGTTGGAGGCGTCGCCAGAGCTCTGGCACACGGTGTTAGGGTCCTGGAAGACGGGGTAAATTATGCAACAGGGAATTTGCCCGGTTGCTCCTTTTCTATCTTCTTGCTTGCTCTTTTGTCGTGCGTCACAGTGCCAGTGTCCGCGGTGGAAGTCAGGAACATTAGTTCTGGCTACTACGCTACCAATGATTGCTCAAACAGCAGCATTACCTGGCAACTCACCAACGCAGTTCTCCACCTTCCCGGCTGCGTCTCATGTGGGAATGACAATGGCACCCTGCGTTGCTGGATACAAGTGACACCTAATGTGGCTGTGAAACATCGCGGCGCACTCACTCACAACCTGCGAACACATGTCGATATGATCGTAATGGCAGCTACGGTCTGCTCGGCCTTGTACGTGGGAGACGCGTGCGGGGCCGTGATGATCGTGTCGCAGGCTCTCATAATATCGCCAGAACGCCACAACTTTACCCAAGAGTGCAACTGTTCCATCTACCAAGGTCATATTACCGGCCACCGCATGGCATGGGACATGATGCTAAATTGGTCACCAACTCTCACCATGCTCCTCGCCTATGCCGCGCGTGTTCCTGAGATGGCCCTTGACATCATCTTCGGCGGCCATTGGGGTGTGGTTTTTGGCTTGGCCTACTTCTCTATGCAGGGAGCGTGGGCCAAGGTCATCGCCATCCTCCTTCTTGTCGCAGGAGTGGACGCATCCACTTCTACCGTTGGTGGAAGTATGGGTGCGAATACCCAGAGATTTACTAGTTTCTTCAGCCTTGGTCCCAGGCAGAAAATCAGTTTGATTAACACCAATGGCAGCTGGCACATAAACCGGACCGCCCTCAATTGCAATGACAGCTTGAACACGGGTTTCATCGCTTCCCTGTTCTACACCAATAACTTCAACAGTTCTGGCTGTCCCGAGCGCTTGTCTTCCTGCCGTAGGTTGGACGATTTCCGCATCGGGTGGGGAGCCTTGGAATACGAGACTAATGTCACCAACGATGAGGACATGAGGCCGTACTGCTGGCATTACCCTCCAAAGCCTTGCGGTATCGTCCCGGCTAGGACAGTTTGCGGGCCAGTCTACTGTTTCACCCCTAGCCCTGTTGTCGTGGGTACCACTGACAGAGAGGGCGTGCCCACCTATAGTTGGGGGGAAAATGAGACCGACGTCTTCTTGCTAAATAGCACAAGACCCCCGCAAGGAGCTTGGTTCGGCTGCACCTGGATGAACGGGACTGGGTTCACTAAGACATGCGGCGCACCACCTTGCCGCATTAGGAGGGACCACAACAGCACCCTCGATCTATTGTGCCCTACAGACTGTTTTAGGAAGCACCCAGACTCTACCTACCTTAAGTGCGGAGCAGGGCCTTGGTTGACCCCCAAATGCCTGGTGGACTACCCATATAGATTGTGGCATTATCCGTGCACTGTGAATTTCACCATCTTCAAAGTGCGGATGTATGTTGGGGGGGTAGAACATCGATTGTCCGCAGCATGCAACTTCACGCGCGGGGATCGCTGCAGGTTGGAGGATAGGGACAGGGGTCAGCAGAGTCCACTGCTGCACTCCACCACTGAGTGGGCGGTATTGCCATGCTCTTTCTCTGATTTGCCGGCGCTGTCTACTGGTCTATTGCACCTCCACCAAAACATCGTGGACGTGCAGTATCTCTATGGACTCACTCCGGCCATCACAAAATACATCGTGAAGTGGGAATGGGTGGTCCTCCTTTTCTTGTTGTTGGCGGACGCTAGGGTCTGCGCGTGCCTTTGGATGCTCATCATACTGGGCCAGGCCGAGGCGGCGCTTGAGAAGCTCATCATCCTGCATTCTGCCAGTGCTGCCAGTGCCAATGGGCCGCTGTGGTTCTTCATCTTCTTTATAGCGGCCTGGTATTTAAAGGGCAGGGTGGTCCCCATGGCCACGTACTCTGTTCTTGGCCTGTGGTCTTTCCTTCTTTTGGTCCTGGCTCTGCCACAGCAGGCTTATGCCCTGGACACTACTGAGCAAGGGCAGCTGGGGCTGGTCATGTTAGCAATCTTATCCATCTTTACGCTCACCCCAGCCTACAAGACTCTCCTGAGCCGCTCGGTGTGGTGGCTGTCCTACATGCTGGTCTTAGCCGAAGCTCAGATTCAGCAGTGGGTTCCCCCCCTGGAGGCCCGAGGGGGGCGTGATGGGATTATCTGGGTAGCTGTCATTCTGCACCCACGTCTTGTGTTTGAGATCACGAAGTGGTTATTAGCAGTCCTGGGGCCTGCCTACCTCCTTAGAGCGTCCCTGCTACGGGTGCCATACTTTGTGAGGGCTCACGCCCTGTTACGAGTGTGCACCCTGGTGAGACACCTCGCAGGAGCTAAGTACATCCAGATGCTGTTGATCACCATAGGCAGGTGGACCGGCACTTACATCTATGACCACCTCTCCCCCTTATCAACTTGGGCAGCCCAAGGTTTGCGGGATCTGGCAGTCGCTGTGGAGCCTGTGGTATTCAGCCCGATGGAGAAGAAGGTCATCGTGTGGGGGGCTGAGACAGTGGCGTGCGGAGATATCCTGCATGGCCTCCCGGTTTCCGCGAGGCTAGGTAGGGAAATTCTGCTCGGCCCTGCCGACGGCTACACCTCCAAGGGGTGGAGACTCCTAGCTCCCATCACTGCTTATACCCAGCAGACTCGCGGTATCCTGGGTGCCATCGTGGTCAGTCTGACGGGCCGCGATAGAAATGAGCAGGCTGGGCAGGTCCAGGTTCTGTCCTCCGTCACACAATCTTTCTTGGGGACATCTATTTCAGGGGTCCTCTGGACAGTATATCATGGAGCTGGCAACAAAACCTTAGCTGGCCCCAAAGGACCAATTACTCAGATGTACACCAGCGCAGAGGGAGACCTCGTAGGATGGCCTAGCCCCCCCGGGACTAAGTCCTTAGACCCCTGTACCTGTGGGGCCGTGGACCTCTACCTGGTCACCCGAAACGCTGATGTCATTCCAGTCCGGAGGAAAGATGACCGGCGGGGCGCATTACTATCGCCAAGGCCCCTCTCAACTCTCAAGGGATCATCAGGCGGACCCGTGCTCTGCTCCAGAGGGCACGCCGTGGGCTTGTTCAGAGCGGCCGTGTGCGCCAGGGGTGTAGCTAAGTCTATTGACTTCATCCCTGTCGAATCTCTTGACATAGTCGCACGGTCACCCAGCTTCTCTGACAACAGCACGCCGCCAGCCGTGCCTCAGACTTACCAAGTGGGCTACCTGCACGCACCAACAGGCAGTGGAAAGAGCACCAAGGTCCCCGCCGCTTATGCCAGTCAGGGGTATAAAGTACTTGTACTAAACCCCTCTGTCGCGGCCACACTTGGTTTTGGGGCCTACATGTCCAAAGCCCACGGGATCAACCCCAACATCAGAACTGGAGTACGGACTGTGACCACCGGAGACTCTATTACCTACTCCACTTATGGCAAGTTCCTCGCAGACGGAGGCTGCTCGGCTGGCGCCTATGACGTCATCATATGCGATGAATGCCACTCAGTGGATTCCACTACCATCCTTGGCATTGGAACAGTCCTTGACCAGGCTGAGACCGCAGGCGCTAGGCTGGTGGTCTTGGCCACGGCCACACCTCCTGGTTCAGTGACAACCCCCCACAGTAACATCGAGGAGGTGGCTCTCGGTCATGAAGGCGAGATCCCGTTTTACGGCAAGGCTATCCCCCTAGCTTACATCAAGGGGGGTAGACACCTGATCTTTTGTCATTCAAAAAAGAAATGCGATGAGCTCGCAGCTGCCCTTCGGGGCATGGGTGTCAACGCCGTTGCTTATTACAGGGGTCTCGACGTCTCTGTTATACCAACTCAAGGAGACGTGGTGGTCGTCGCCACCGACGCCCTAATGACTGGATACACCGGTGACTTTGATTCTGTCATCGACTGCAACACTGCAGTCACTCAGGTTGTTGACTTTAGTCTGGACCCAACCTTTACCATTACTACTCAAACCGTCCCTCAGGATGCTGTCTCCCGAAGCCAACGTAGAGGGAGAACTGGGAGGGGACGACTGGGCATTTACAGGTATGTCTCGTCAGGAGAGAGGCCGTCTGGGATGTTCGACAGCGTAGTGCTCTGTGAGTGCTATGATGCCGGGGCAGCCTGGTACGAGCTCACGCCTGCTGAGACTACAGTGAGACTCCGGGCTTACTTCAACACGCCCGGCCTGCCCGTGTGTCAAGACCACCTGGAATTCTGGGAGGCGGTCTTCACGGGTCTCTCACACATCGATGCCCACTTCCTCTCCCAGACGAAGCAGGCAGGAGACAATTTTGCGTACCTGGTGGCCTATCAGGCCACAGTGTGCGCTAGGGCAAAGGCCCCTCCTCCTTCGTGGGACGTGATGTGGAAGTGTCTGACTAGGCTCAAACCTACACTGACTGGTCCTACCCCCCTCCTGTACCGCTTAGGTGCCGTGACCAATGAGGTTACCCTGACGCATCCCGTGACAAAGTATATCGCCACGTGCATGCAAGCTGACCTTGAGATCATGACGAGCACATGGGTCCTGGCAGGGGGGGTGTTAGCCGCCGTGGCGGCTTACTGCCTGGCAACCGGCTGCGTTTCCATCATTGGCCGCCTACACCTGAATGACCAGGTAGTTGTGGCCCCCGATAAGGAAATCTTATATGAGGCCTTTGATGAGATGGAAGAGTGCGCCTCCAAAGCCGCCCTCATTGAGGAAGGGCAGCGGGTGGCGGAGATGCTGAAGTCCAAAATACAAGGCCTCTTACAACAGGCCACAAGACAGGCCCAAGACATACAGCCAGCCATACAGTCATCATGGCCCAAGCTCGAACAATTTTGGGCCAAACACATGTGGAACTTCATCAGTGGCATACAGTACCTGGCGGGACTCTCTACCCTACCGGGAAATCCTGCAGTGGCATCGATGATGGCTTTCAGCGCCGCATTGACTAGCCCACTGCCCACCAGCACCACCATCCTCTTGAACATCATGGGGGGATGGTTGGCCTCTCAGATAGCCCCCCCTGCCGGAGCCACTGGCTTCGTTGTCAGTGGTCTAGTGGGGGCGGCCGTCGGAAGCATAGGCCTGGGTAAGATATTGGTGGATGTCTTGGCTGGGTATGGCGCAGGTATTTCGGGGGCCCTCGTAGCTTTTAAGATCATGAGCGGCGAGAAGCCCTCGGTAGAAGATGTTGTCAATCTCCTGCCCGCCATCTTGTCTCCTGGTGCTTTGGTAGTGGGGGTCATCTGCGCAGCAATCTTGCGCCGCCACGTTGGTCAGGGGGAGGGGGCAGTCCAGTGGATGAACAGGCTGATTGCCTTCGCCTCCAGAGGAAACCATGTTGCCCCTACCCACTACGTGGCAGAGTCTGACGCTTCGCAGCGCGTGACGCAAGTGTTGAGCTCACTCACAATTACCAGCTTACTTAGGAGGCTACATACCTGGATCACTGAAGATTGCCCAGTCCCGTGCTCGGAGTCTTGGCTCCGGGACATTTGGGATTGGGTTTGTTCCATCCTCACAGACTTTAAGAACTGGCTGTCCTCAAAACTGCTCCCTAAAATGCCCGGCCTTCCCTTTATCTCTTGCCAGAAGGGATACAAGGGTGTATGGGCTGGCACGGGAGTCATGACCACTCGGTGTCCATGCGGAGCAAACATCTCGGGCCATGTCCGCTTGGGTACCATGAAAATAACGGGCCCGAAGACCTGCTTGAACCTTTGGCAGGGGACCTTTCCCATCAATTGTTACACAGAAGGGCCGTGCGTGCCAAAACCCCCTCCTAATTATAAGACCGCAATTTGGAGGGTGGCAGCGTCGGAGTACGTTGAGGTCACGCAGCATGGCTCTTTCTCGTACGTAACGGGGTTAACCAGTGACAACCTTAAGGTCCCCTGTCAGGTTCCAGCTCCAGAATTCTTTTCTTGGGTGGATGGGGTACAGATACACCGGTTCGCCCCCACTCCAGGTCCCTTCTTTCGGGATGAGGTAACGTTTTCCGTAGGCCTCAATTCCTTTGTGGTTGGCTCTCAGCTCCCTTGTGACCCCGAGCCAGACACGGAGGTACTAGCCTCCATGTTGACAGACCCGTCCCACATTACAGCGGAGGCGGCAGCTAGGCGATTGGCCAGGGGATCTCCCCCCTCACAGGCCAGCTCCTCGGCGAGCCAGCTCTCAGCCCCGTCTTTGAAGGCTACCTGTACCACCCACAAGATGGCATATGACTGTGACATGGTGGACGCTAACCTTTTCATGGGAGGCGATGTGACCCGAATTGAGTCCAGCTCGAAGGTGATTGTTCTCGACTCCCTCGATTCCATGACTGAGGTAGAGGACGACCGCGAGCCTTCCATACCATCGGAGTACTTGATCAGGAGGAAAAAGTTCCCATCGGCATTACCCCCCTGGGCCCGCCCAGACTACAATCCTCCTGTAATCGAGACATGGAAGAGGCCGGGTTATGAACCACCCACTGTCCTAGGCTGTGCCCTTCCTCCCACACCTCAAGTGCCAGTGCCCCCACCTCGGAGGCGCCGCACCAAGGTCCTGACTCAGGACAATGTGGAGGGAGCCCTCAGGGAGATGGCGGACAAAGTGTTCAGCCCTCCCCAAGGTCACGATGACTCCGGTCACTCCACTGGAGTGGATACCGGGGGAGACAGCGTCCAACAGCCCTCTGACGAGACTGCCGATTCGGAAGCAGGATCACTGTCCTCCATGCCTCCCCTTGAGGGAGAGCCGGGAGACCCTGATCTGGAGTTTGAGCCGGCTAGATCCGCTCCCTCCTCCGAGGGGGAGTGCGAGGTCGCCGATTCGGACTCCAAGTCATGGTCCACAGTCTCTGATCAAGAGGATTCTGTCATCTGTTGCTCCATGTCATACTCCTGGACAGGGGCCCTCATAACACCATGCGGGCCCGAAGAGGAGAAGTTGCCAATTAACCCTCTGAGCAATTCGCTCATGCGGTACCATAACAAGGTATACTCCACAACTTCGCGGAGCGCGTCTCTGAGGGCAAAGAAGGTGACTTTTGACAGAGTACAGATACTGGACACATACTATGACTCAGTCTTGCAGGACGTTAAGCGGGCCGCCTCTAAGGTTAGTGCGAGGCTCCTCTCAATAGAGGAAGCCTGCGCTCTGACTCCGCCCCACTCCGCCAAATCGCGATACGGATTTGGGGCAAAAGAGGTGCGCAGCTTGTCCAGGAGGGCCGTCAACCACATCCAGTCCGTGTGGGAGGACCTCCTGGAAGACCAACATACTCCAATTGAAACAACCATCATGGCCAAAAATGAGGTGTTCTGTGTTGATCCCGCCAAAGGTGGGAAGAAGCCAGCTCGCCTCATCGTATACCCAGACCTGGGGGTCAGGGTGTGCGAAAAGATGGCCTTATATGACATTGCACAAAAGCTTCCCAAGGCAATAATGGGATCATCCTATGGGTTCCAATACTCTCCTGCAGAACGGGTCGATTTTCTCCTCAAAGCTTGGGGAAGCAAGAAGGACCCAATGGGGTTCTCATATGACACCCGCTGCTTTGACTCAACCGTCACGGAGAGGGACATAAGAACAGAGGAATCCATATACCAGGCTTGTTCCCTGCCTGAAGAGGCCAGAACTGCCATACACTCGCTCACTGAGAGACTTTACGTAGGAGGGCCCATGATAAACAGCAAAGGCCAATCCTGCGGTTACAGGCGTTGCCGCGCAAGTGGCGTTTTCACAACCAGCATGGGGAACACCATGACATGCTACATCAAGGCCCTTGCGGCGTGCAAAGCTGCAGGGATCGTGGACCCTATTATGCTGGTGTGTGGAGACGACCTGGTCGTCATCTCAGAGAGCCAGGGCAACGAGGAGGACGAGCAGAACCTGAGAGCTTTCACGGAGGCTATGACCAGGTATTCAGCCCCTCCCGGTGACCTTCCCAGACCGGAATACGACTTGGAGCTTATAACATCATGCTCCTCAAACGTGTCGGTTGCGCTGGACCCGCGGGGTCGCCGCCGATACTACCTAACTAGAGACCCTACCACTCCAATCTCCCGAGCTGCTTGGGAAACAGTAAGACACTCCCCTGTCAATTCTTGGCTGGGCAACATCATCCAGTACGCCCCTACAATCTGGGTCCGGATGGTCATATTGACCCACTTCTTCACCATACTACTGGCCCAGGACACTCTGAACCAAAATCTCAACTTTGAGATGTACGGGGCAGTGTATTCGGTCAATCCATTAGACCTACCGGCCATAATTGAAAGGCTACATGGGCTTGATGCATTTTCACTGCACACATACTCTCCTCACGAACTCTCACGGGTGGCAGCAACTCTCAGAAAACTTGGAGCGCCTCCCCTTAGAGCGTGGAAGAGTCGGGCGCGTGCTGTGAGAGCATCACTCATCGCCCAGGGTGGGAGAGCGGCCATTTGTGGCCGCTACCTCTTCAACTGGGCGGTGAAAACAAAGCTCAAACTCACTCCATTGCCCGAGGCGAGCCGCCTGGATTTATCCGGGTGGTTCACCGTGGGCGCCGGCGGGGGCGACATCTTTCACAG

>PAT2

CACTCCCCTGTGAGGAACTACTGTCTTCACGCAGAAAGCGTCTAGCCATGGCGTTAGTATGAGTGTCGTACAGCCTCCAGGCCCCCCCCTCCCGGGAGAGCCATAGTGGTCTGCGGAACCGGTGAGTACACCGGAATTACCGGAAAGACTGGGTCCTTTCTTGGATAAACCCACTCTATGTCCGGTCATTTGGGCGTGCCCCCGCAAGACTGCTAGCCGAGTAGCGTTGGGTTGCGAAAGGCCTTGTGGTACTGCCTGATAGGGTGCTTGCGAGTGCCCCGGGAGGTCTCGTAGACCGTGCATCATGAGCACAAATCCTAAACCTCAAAGAACGACCAAAAGAAACACAAACCGCCGCCCACAGGACGTTAAGTTCCCGGGTGGCGGCCAGATCGTTGGCGGAGTTTACTTGTTACCGCGCAGGGGCCCCAGGTTGGGTGTGCGCGCGACAAGGAAGACTTCTGAGCGATCCCAGCCGCGTGGGAGACGCCAGCCCATCCCGAAAGATCGGCGCTCCACCGGCAAGTCCTGGGGAAAGCCAGGATATCCTTGGCCCCTGTACGGAAACGAGGGCTGCGGCTGGGCAGGTTGGCTCCTGTCCCCCCGCGGTTCCCGTCCTACCTGGGGCCCCACTGACCCCCGGCACAGATCACGCAATTTGGGCAGAGTCATCGACACCATTACGTGTGGTTTTGCCGACCTCATGGGGTACATCCCTGTCGTAGGCGCCCCGGTTGGAGGCGTCGCCAGAGCTCTGGCACACGGTGTTAGGGTCCTGGAAGACGGGATAAATTATGCAACAGGGAATCTGCCTGGTTGCTCTTTCTCTATCTTCTTACTTGCTCTTCTGTCATGCGTCACGGTGCCAGTATCTGCGGTGGAGGTCAGGAACATCAGTTCTAGCTACTACGCCACCAATGATTGCTCGAACAACCACATTACTTGGCAACTCACCAACGCAGTTCTCCATCTTCCCGGATGCGTCCCATGTGAGAATGACAATGGCACCCTGCGATGCTGGATACAAGTGACACCCAATGTGGCTGTGAAACACCGCGGTGCGCTCACTCACAACCTGCGGACACATGTCGATATGATCGTAATGGCAGCTACGGTCTGCTCGGCCTTGTATGTGGGAGACATGTGCGGGGCCGTGATGATTGTGTCGCAGGCTTTCATAATATCACCAGAGCGCCACAACTTCACCCAAGAGTGTAACTGTTCCATCTACCAAGGCCATATCACCGGCCATCGCATGGCATGGGACATGATGCTAAATTGGTCACCAACTCTCACCATGGTCCTTGCCTATGCAGCTCGTGTCCCTGAGCTAGCCCTTGAAGTTGTCTTCGGCGGCCATTGGGGTGTGGTGTTTGGCCTGGCCTATTTCTCTATGCAAGGAGCGTGGGCCAAGGTCGTCGCCATCCTCCTCCTTGTCGCGGGGGTGGATGCAAACACTGTAGTCACCGGTGGACAAGCGGGTCGTGATGCCTACAGGCTTGCCAATCTCTTTTCCTTTGGTCCCAAGCAGGAAATCAATTTAATCAACACCAATGGCAGCTGGCACATAAATCGGACCGCCCTCAATTGCAATGACAGCTTGCAAACGGGTTTCATCGCTTCCCTGTTCTACGCCAGAAGCTTTAACAGTTCTGGCTGCCCCGAGCGCTTGGCTTCCTGCCGTGGGCTGGACGATTTCCGCATCGGGTGGGGGACCTTGGAATACGAGACTAATGTCACCAACGATGAGGACATGAGGCCGTACTGCTGGCATTACCCTCCGAGGCCTTGCGGTATCGTCCCGGCTAGGACAGTTTGCGGGCCAGTCTACTGTTTCACTCCCAGCCCTGTTGTCGTGGGGACCACTGACAGACATGGCGTGCCCACCTATAGTTGGGGGGAAAATGAGACCGACGTCTTTCTGCTGAATAGCACAAGACCCCCGCAAGGAGCTTGGTTCGGCTGCACCTGGATGAACGGGACTGGGTTCACTAAGACATGCGGTGCACCACCTTGCCGCATTAGGAGGGATCACAACAGCACCCTCGATCTATTGTGCCCTACAGACTGTTTTAGAAAGCATCCAGCATCTACTTACCTCAAGTGTGGAGCAGGGCCTTGGTTGACCCCCAGATGCCTAGTAGACTACCCATATAGATTGTGGCATTATCCGTGCACTGTCAATTTTACTATCTTCAAGGTGCGGATGTATGTTGGAGGGGTGGAACATCGATTGTCCGCGGCATGCAACTTTACGCGCGGGGACCGCTGCAGGTTGGATGATAGGGACAGAGGCCAGCAGAGTCCGCTGTTGCATTCCACCACCGAGTGGGCGGTATTGCCATGCACTTTCTCTGACTTACCGGCGCTGTCTACTGGTCTATTGCATCTTCACCAAAACATCGTGGACGTGCAGTACCTCTATGGACTCTCTCCGGCCATCACAAGATACATCGTGAAGTGGGAGTGGGTGGTTCTCCTCTTTTTGTTGCTGGCCGATGCCAGGGTCTGCGCATGCCTTTGGATGCTCATCATATTGGGCCAGGCCGAGGCGGCGCTTGAGAAGCTCATCATCTTGCACTCTGCTAGCGCCGCTAGCGCCAATGGGCCGCTATGGTTCTTCATCTTTTTTGTGGCGGCCTGGTATTTAAAGGGTAGAACAGTCCCCATGGCCACGTACTCTGTTCTTGGCTTGTGGTCTTTCCTCCTTTTGGTCCTGGCCCTGCCACAGCAGGCCTACGCCCTGGACGCCACTGAACAAGGGGAACTGGGGCTGGTCGTGCTAGTAATCTTGTCCATCTTTACTCTCACCCCGGCATATAAGACTCTTCTGAGTCGCTCGGTGTGGTGGCTGTCCTACATGCTGGTCTTAGCCGAAGCTCAGATTCAGCAGTGGGTTCCCCCCCTGGAGGCCCGGGGGGGGCGTGATGGGATCATCTGGCTAGCTGTCATTCTGCACCCACGCCTCGTGTTTGAAGTCACGAAGTGGTTATTAGCAGTTCTGGGGTCCGCCCACCTCCTTAGGGCGTCCCTGCTACGGGTGCCATACTTTGTGAGGGCTCACGCCCTGTTACGAGTGTGTACCCTAGTGAAACACCTTGCAGGAGCTAGGTACATCCAGATGCTGTTGATCACCATAGGCAAGTGGACAGGCACTTACATCTATGACCACCTCTCCCCCTTATCAACTTGGGCAGCCCAGGGTTTGCGGGACTTAGCAGTTGCTGTGGAGCCTGTGGTGTTCAGCCCAATGGAGAAGAAGGTCATTGTGTGGGGGGCTGAGACAGTGGCATGCGGGGATATTCTGCATGGCCTCCCGGTTTCCGCGAGGCTAGGTAGGGAAGTTCTGCTCGGCCCCGCCGATGGCTACACCTCCAAGGGGTGGAGGCTCCTAGCTCCCATCACTGCTTATACCCAGCAGACTCGTGGCCTCCTGGGCGCCATCGTGGTCAGTTTAACGGGCCGCGATAGAAATGAGCAGGCCGGGCAAGTTCAGATTCTATCCTCTGTCACACAATCCTTCTTGGGGACATCTATTTCGGGGGTCCTCTGGACAGTATATCATGGGGCTGGCAACAAGACCTTAGCTGGCCCTAAAGGACCAATTACTCAGATGTATACCAGCGCAGAGGGGGACCTCGTGGGGTGGCCTAGCCCCCCTGGGACTAAGTCTTTAGACCCCTGTACCTGCGGGGCTGTGGACCTCTACCTGGTCACCCGAAACGCTGATGTCATCCCGGTCCGGAGGAAAGATGACCGGCGGGGCGCGCTACTCTCGCCGAGGCCCCTCTCAACTCTCAAAGGATCTTCCGGTGGACCCGTGCTCTGCTCTAGGGGGCATGTCGTGGGGTTATTTAGAGCGGCCGTGTGTGCTAGGGGTGTAGCCAAATCTATTGACTTCATCCCTGTCGAATCTCTCGACATAGTCACGCGATCGCCCAGCTTCTCTGACAACAGCACTCCACCAGCTGTGCCCCAGACTTACCAGGTGGGCTACCTGCACGCTCCAACAGGCAGTGGGAAGAGCACCAAGGTCCCCGCTGCTTACGCCAGTCAGGGGTATAAGGTACTTGTACTAAATCCCTCTGTCGCGGCCACACTTGGTTTTGGGGCCTACATGTCCAAAGCCCACGGAATCAACCCCAACATCAGGACCGGAGTGCGGACTGTGACCACCGGGGACCCTATCACCTACTCCACTTATGGCAAGTTTCTCGCAGACGGAGGCTGCTCGGCTGGCGCCTATGATGTCATCATATGCGACGAATGCCATGCAGTGGATGCCACTACCATTCTTGGCATTGGAACAGTCCTTGACCAGGCTGAGACCGCTGGCGCCAGGCTAGTGGTCTTGGCCACAGCCACACCTCCCGGTTCGGTGACAACTCCCCACAGTAACATAGAGGAGGTGGCTCTTGGTCATGAAGGCGAGATCCCTTTTTACGGCAAGGCTATCCCTCTAGCTTTCATCAAGGGGGGCAGACACCTGATCTTTTGTCACTCAAAAAAGAAATGCGATGAGCTCGCAGCAGCCCTGCGGGGCATGGGTGTCAACGCCGTTGCTTACTATAGGGGTCTTGACGTCTCTGTTATACCAACTCAAGGAGATGTGGTGGTTGTTGCCACCGACGCCCTAATGACTGGATATACCGGTGACTTTGACTCTGTCATCGACTGCAACGTTGCAGTCACTCAGATTGTTGACTTTAGCCTAGACCCAACTTTTAGCATCACCACTCAAACTGTCCCTCAGGATGCTGTCTCCCGTAGCCAACGTAGGGGGAGAACTGGGAGGGGACGATTGGGCATTTATAGGTATGTCTCGTCGGGTGAGAGGCCGTCTGGGATGTTCGACAGCGTAGTGCTCTGCGAGTGCTATGATGCCGGGGCAGCCTGGTACGAACTCACGCCTGCTGAGACTACAGTGAGACTCCGGGCTTACTTCAACACGCCTGGTTTACCCGTGTGCCAGGACCATCTGGAATTCTGGGAGGCAGTCTTCACAGGTCTCACACACATCGATGCCCACTTCCTCTCCCAAACGAAGCAGGGAGGCGACAACTTTGCGTATCTGGTGGCTTATCAGGCCACAGTATGCGCTAGGGCAAAAGCCCCTCCTCCTTCGTGGGATGCGATGTGGAAGTGTCTAACTAGGCTTAAACCTACACTGAATGGTCCTACCCCCCTCCTGTACCGCTTGGGTGCTGTGACCAACGAGGTTACCCTGACGCACCCCGTGACGAAATATATCGCCACGTGCATGCAGGCTGACCTTGAGATCATGACAAGCACATGGGTTCTGGCAGGGGGGGTGCTAGCCGCCGTGGCGGCTTACTGCCTGGCAACCGGCTGCGTTTCCATCATTGGCCGCTTGCACCTGAATGACCAGGTAGTCGTGGCCCCTGACAAAGAAATCCTGTATGAGGCCTTTGATGAGATGGAAGAGTGCGCCTCCAAAGCCGCCCTCATTGAGGAAGGGCAGCGGATCGCGGAGATGCTAAAGTCCAAGATACAAGGCCTCTTACAACAGGCCACGAGACAGGCCCAAGACATACAGCCAGCCATACAGTCATCATGGCCCAAGCTTGAACAATTCTGGGCCAAACACATGTGGAATTTCATCAGTGGCATACAGTACCTGGCGGGACTCTCCACTTTACCGGGAAATCCCGCAGTGGCATCAATGATGGCTTTCAGTGCTGCATTGACTAGCCCACTGTCCACCAGCACCACCATCCTCTTGAACATCATGGGGGGATGGCTGGCCTCTCAGATTGCCCCCCCTGCCGGAGCCACTGGTTTTGTTGTCAGTGGTTTAGTAGGGGCGGCCGTCGGAAGCATAGGCCTGGGTAAGATATTGGTGGACGTTTTGGCCGGGTACGGCGCAGGTATTTCAGGGGCTCTCGTAGCTTTTAAGATCATGAGCGGCGAGAAACCCTCAGTGGAGGATGTTGTAAATCTCCTGCCTGCAATCTTGTCTCCTGGTGCTTTGGTAGTGGGAGTCATCTGTGCGGCAATCTTACGCCGCCACGTCGGCCAGGGGGAGGGGGCAGTTCAATGGATGAACAGGCTAATCGCCTTTGCCTCCAGAGGAAACCATGTTGCCCCCACCCATTACGTGGCAGAGTCTGACGCTTCGCAGCGCGTGATGCAAGTGTTGAGCTCACTCACAATTACCAGCTTACTTAGGAGCCTTCATACCTGGATCACTGAAGATTGCCCAGTCCCGTGCTCGGAGTCTTGGCTCCGGGACATTTGGGATTGGGTCTGCTCGATCCTCACAGACTTTAAGAACTGGCTGTCCTCAAAACTGCTCCCCAAATTGCCTGGCCTTCCCTTTATCTCTTGTCAAAAGGGATACAAGGGTGTGTGGGCTGGCACGGGAGTCATGACCACTCGGTGTCCTTGCGGAGCAACCATCTCGGGCCATGTCCGCATGGGCACCATGAAAATAACAGGCCCGAAGACCTGCTTGAACTTATGGCAGGGGACCTTCCCCATTAATTGCTACACAGAAGGGCCTTGCGTGCCAAAACCTCCTCCTAATTATAAGACTGCAATTTGGAGGGTGGCAGCGTCGGAGTACGTTGAGGTCACGCAGCATGGCTCTTTCTCGTACGTAACGGGGTTAACCAGTGACAATCTTAAGGTCCCTTGCCAGGTACCAGCTCCAGAATTCTTCTCTTGGGTGGACGGGGTGCAGATACACCGATTCGCCCCCACTCCAGGTCCCTTCTTTCGGGATGAGGTAACGTTCTCCGTAGGCCTCAATTCCTTTGTGGTCGGCTCTCAGCTCCCTTGTGACCCTGAGCCAGACACGGAGGTGCTAGCCTCCATGTTAACAGATCCGTCCCACATTACAGCGGAGGCGGCAGCTAGGCGACTGGCTAGGGGGTCTCCCCCCTCACAGCCCAACTCGTCAGCAAGCCAACTCTCCGCCCCGTCTTTGAAGGCTACCTGTACCACCCATAAGATGGCATATGACTGTGACATGGTAGATGCTAACCTTTTCATGGGAGGCGATGTGACCCGGATTGAGTCCAGCTCGAAGGTGGTTGTTCTCGACTCCCTCGATTCTATGATTGAGGTAGAGGACGATCGTGAGCCTTCTATACCATCAGAGTACTTAATTAGGAGGAAAAAGTTTCCACCGGCACTACCTCCCTGGGCCCGTCCAGACTATAATCCTCCCGTGATCGAGACATGGAAGAGGCCGGACTATGAACCACCCACTGTCTTAGGTTGTGCCCTTCCTCCCACACCTCAAGTGCCAGTGCCCCCACCTCGGCGACGCCGCGCCAAGGTCCTGACTCAGGACAATGTGGAGGAGGCCCTCAGGGAGATGGCGAACAAAGCGTTCAGCCCTCCCCAAGATTGCAATGACTCCGGTCACTCCACTGGAGTGGATACCGGGGGAGACAGCGTCCAGCAGCCCTCTGACGAGACTGCCGCTTCGGAAACAGGATCTCTGTCTTCCATGCCTCCCCTTGAGGGAGAGCCGGGGGACCCTGATCTGGAGCTTGAGCCAGCTAGATCCGCTCCCCCTTCCGAGGGGGAGTGTGAGGTCGCTGATTCGGACTCCAAGTCATGGTCCACAGTCTCTGATCAAGAGGATTCTGTCATCAGTTGTTCCAAGTCATACTCCTGGACAGGGGCTCTCATAACACCATGTTGGCCCGAGGAGGAGAAGTTGCCAATCAACCCTCTGAGCAATTCGCTCATGCGGTTCCATAACAAGGTGTACTCCACAACCTCGCGGAGTGCCTCTTTGAGGGCAAAGAAGGTAACTTTTGACAGGGTGCAGGTACTGGACACATACTATGACTCAGTCTTGCAGGACGTCAAGCGGGCCGCCTCTAAGGTTAGTGCGAGGCTCCTCTCAATAGAGGAAGCCTGCGCGCTGACCCCGCCCCACTCCGCCAAATCGCGGTACGGATTTGGGGCGAAAGAGGTGCGCAGCTCGTCCAGGAGGGCCGTCAACCACATCCAGTCCGTGTGGGAGGACCTCCTGGAAGACCAACATACTCCAATTCAGACAACCATCATGGCCAAAAACGAGGTGTTCTGTGTTGATCCCGCTAAAGGCGGGAAGAAGCCAGCTCGCCTCATCGTATTCCCTGACCTTGGGGTCAGGGTGTGCGAAAAGATGGCCTTATATGACATTGCACAAAAACTTCCTAAGGCAATAATGGGATCATCCTATGGGTTCCAATACTCTCCTGCAGAACGGGTCGATTTTCTCCTCAAAGCTTGGGGAAGCAAGAAGGACCCAATGGGGTTCTCATATGACACCCGCTGCTTTGACTCAACCGTCACGGAGAGAGATATAAGAACAGAGGAATCCATATACCAGGCTTGTTCCCTGCCTGAAGAGGCTAGAGTTGCCATACACTCGCTCACTGAGAGACTTTACGTAGGAGGGCCCATGATAAACAGCAAGGGCCAGTCCTGCGGCTACAGGCGTTGCCGCGCAAGCGGCGTTTTCACAACCAGCATAGGGAACACCATGACGTGTTATATCAAAGCCCTTGCGGCGTGCAAAGCTGCAGGAATCGTGGACCCTGTCATGCTGGTGTGTGGAGACGACCTGGTCATTATCTCAGAGAGCCAGGGCAACGAGGAGGACGAGCGAAACCTGAGAGCTTTCACGGAGGCTATGACCAGGTATTCAGCCCCTCCCGGTGACCCTCCCAGGCCGGAATATGACTTGGAGCTTATAACATCCTGCTCCTCAAACGTGTCGGTAGCGCTGGACCCTCGAGGTCGCCGCCGATACTACCTAACCAGAGACCCTACCACTCCAATCACCCGAGCTGCTTGGGAAACAGTAAGACACTCCCCTGTCAATTCTTGGCTGGGCAACATCATCCAGTACGCCCCTACAATCTGGGTCCGGATGGTCATGATGACCCACTTCTTCTCCATACTACTGGCCCAGGACACCCTGAACCAAAATCTCAACTTTGAGATGTATGGGGCAGTATATTCGGTCAATCCATTAGACCTACCAGCCATAATTGAAAGGTTACATGGGCTTGATGCTTTCTCATTGCACACATACTCTCCTCACGAACTCTCACGGGTGGCAGCAACTCTCAGAAAACTTGGAGCGCCTCCCCTTAGAGCGTGGAAGAGTCGGGCGCGTGCTGTGAGAGCATCACTCATCGGCCAGGGCGGGAGGGCGGCCACTTGCGGCCGTTACCTCTTTAACTGGGCGGTGAAGACAAAGCTCAAACTCACTCCATTGCCCGAGGCGACCCGCCTGGATTTATCCGGGTGGTTCACCGTGGGCGCCGGCGGGGGCGACATCTATCACAG
