# Supplementary figures and images for: The First Complete Genome Sequences of Hepatitis C Virus Subtype 2b from Latin America: Molecular Characterization and Phylogeographic Analysis
Source: Viruses. 2019 Oct 31;11(11):1000. doi: 10.3390/v11111000 (PMC6893431; doi:10.3390/v11111000)

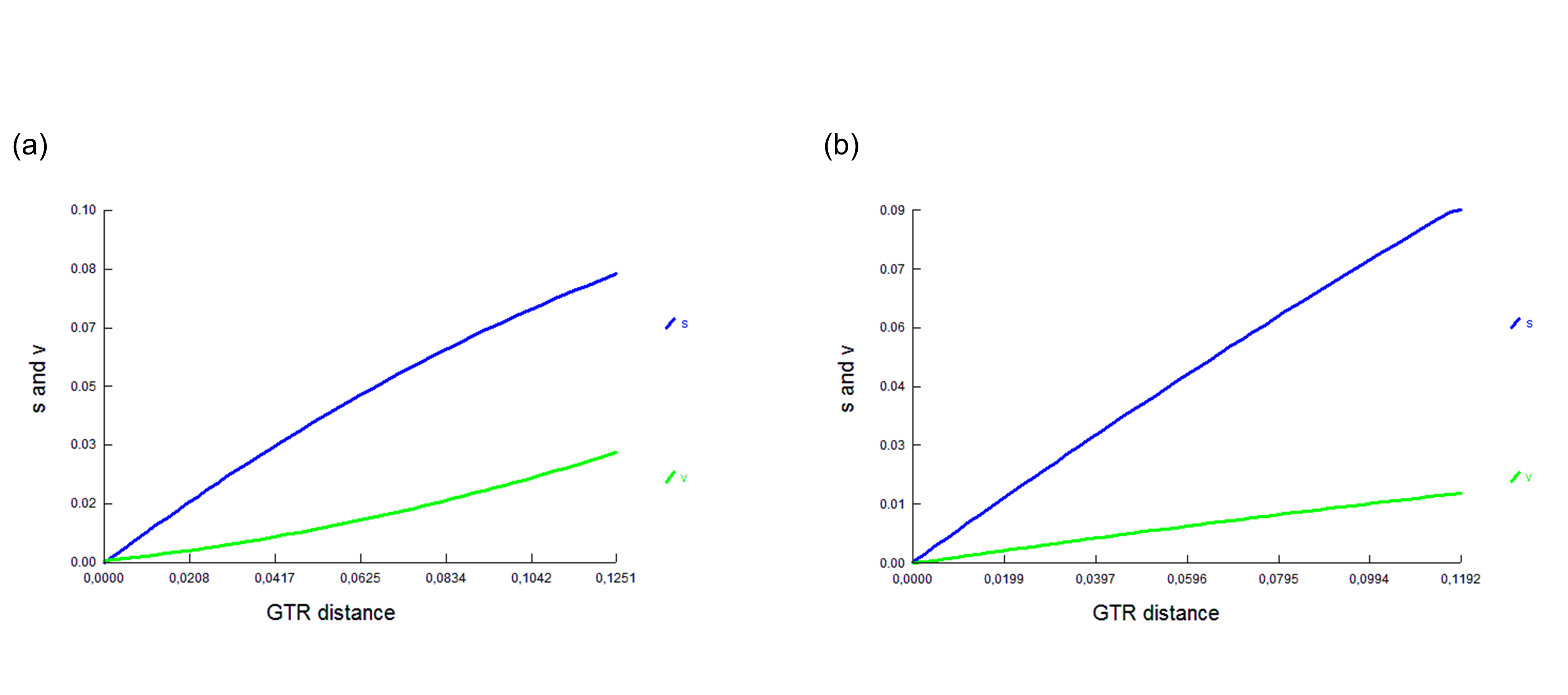

Supplement: Supplementary file 1 [file viruses-11-01000-s001.zip › Supplementary Fig 1.tif]

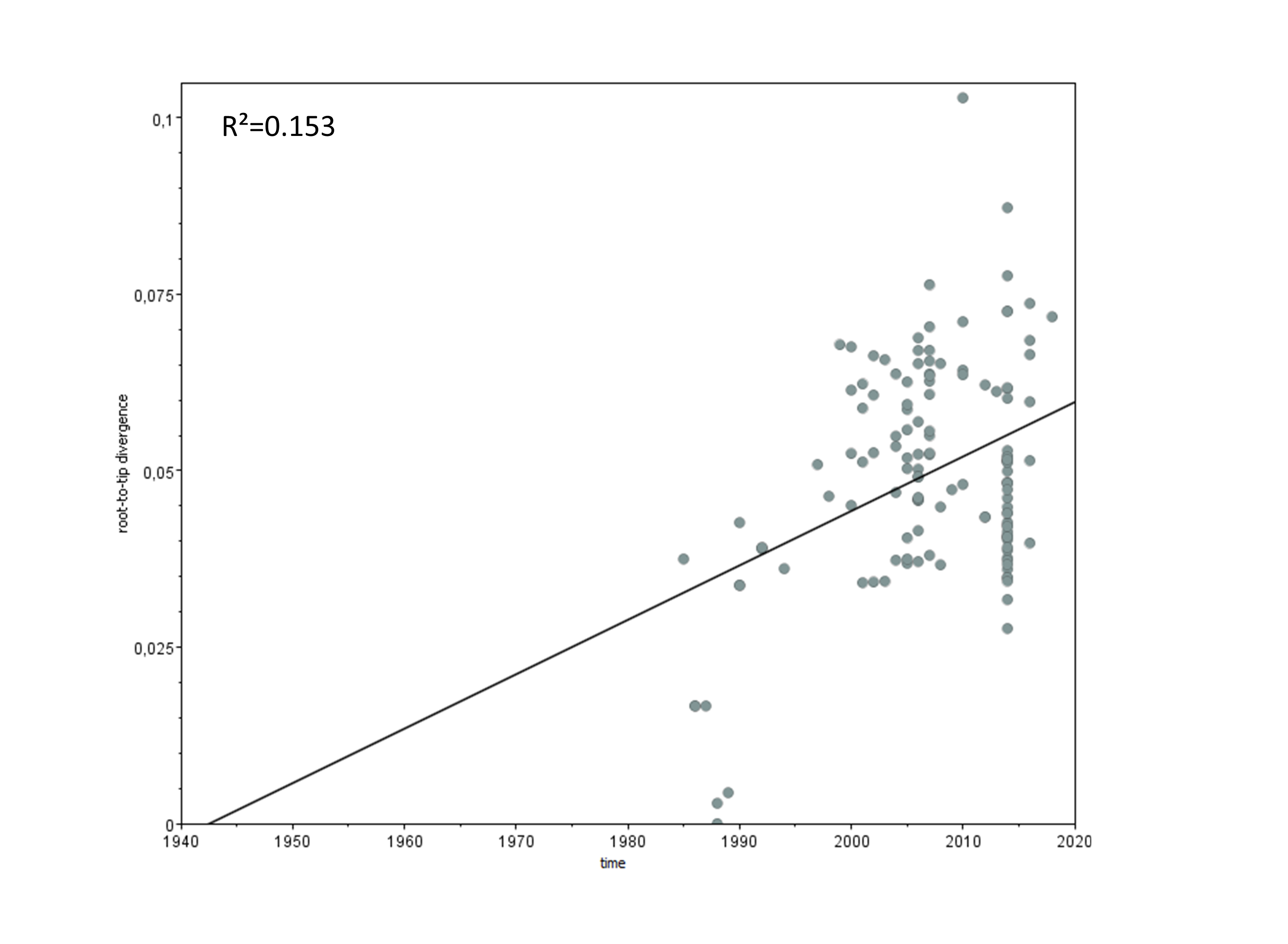

Supplement: Supplementary file 1 [file viruses-11-01000-s001.zip › Supplementary Fig 2.tif]
